# Prospective validation of a mobile health application for blood pressure management in patients with hypertensive disorders of pregnancy: study protocol for a randomized controlled trial

**DOI:** 10.1186/s13063-024-08200-y

**Published:** 2024-07-02

**Authors:** Ju-Seung Kwun, Jina Choi, Yeonyee E. Yoon, Hong-Mi Choi, Jee Yoon Park, Hyeon Ji Kim, Min Jung Lee, Bo Young Choi, Sooyoung Yoo, Jung-Won Suh

**Affiliations:** 1 Department of Internal Medicine, Cardiovascular Center, Seoul National University Bundang Hospital, 82, Gumi-Ro 173 Beon-Gil, Bundang-Gu, Seongnam-Si, Gyeonggi-Do, 13620 Korea; 2 Department of Internal Medicine, Seoul National University, Seoul, Korea; 3 Department of Obstetrics and Gynecology, Seoul National University College of Medicine, Seoul National University Bundang Hospital, Seongnam, Korea; 4 Healthcare ICT Research Center, Office of eHealth Research and Businesses, Seoul National University Bundang Hospital, Seoul, Korea

**Keywords:** Hypertensive disorder of pregnancy, Mobile healthcare, Electronic medical records, Randomized controlled trial

## Abstract

**Background:**

Hypertensive disorders of pregnancy (HDP) pose significant risks to both maternal and fetal health, contributing to global morbidity and mortality. Management of HDP is complex, particularly because of concerns regarding potential negative effects on utero-placental circulation and limited therapeutic options due to fetal safety. Our study investigates whether blood pressure monitoring through a mobile health (mHealth) application can aid in addressing the challenges of blood pressure management in pregnant individuals with HDP. Additionally, we aim to assess whether this intervention can improve short-term maternal and fetal outcomes and potentially mitigate long-term cardiovascular consequences.

**Methods:**

This prospective, randomized, single-center trial will include 580 pregnant participants who meet the HDP criteria or who have a heightened risk of pregnancy-related hypertension due to factors such as multiple pregnancies, obesity, diabetes, or a history of HDP in prior pregnancies leading to preterm birth. Participants will be randomized to either the mHealth intervention group or the standard care group. The primary endpoint is the difference in systolic blood pressure from enrollment to 1 month after childbirth. The secondary endpoints include various blood pressure parameters, obstetric outcomes, body mass index trajectory, step counts, mood assessment, and drug adherence.

**Conclusions:**

This study emphasizes the potential of mHealth interventions, such as the Heart4U application, to improve blood pressure management in pregnant individuals with HDP. By leveraging technology to enhance engagement, communication, and monitoring, this study aims to positively impact maternal, fetal, and postpartum outcomes associated with HDP. This innovative approach demonstrates the potential of personalized technology-driven solutions for managing complex health conditions.

**Trial registration:**

ClinicalTrials.gov NCT05995106. Registered on 16 August 2023.

**Supplementary Information:**

The online version contains supplementary material available at 10.1186/s13063-024-08200-y.

## Introduction

### Background and rationale {6a}

Hypertensive disorders of pregnancy (HDP), encompassing chronic hypertension, gestational hypertension, preeclampsia, and preeclampsia superimposed on chronic hypertension, remains a significant contributor to maternal and fetal morbidity and mortality worldwide [1, 2]. Moreover, a history of HDP heightens the risk for future cardiovascular events [3, 4]. With the recent surge in the prevalence of advanced maternal age, there has been a simultaneous increase in the incidence of HDP, particularly in older expectant mothers [5, 6].

Effectively managing HDP not only addresses immediate maternal and fetal health but also has the potential to impact long-term adverse cardiovascular outcomes for the mother. However, the availability of pharmaceutical agents for controlling HDP is limited due to concerns regarding fetal safety [7, 8]. Consequently, active monitoring of both blood pressure and obstetric parameters plays a critical role in achieving optimal control and mitigating potential complications associated with HDP [9].

**Fig. 1:**
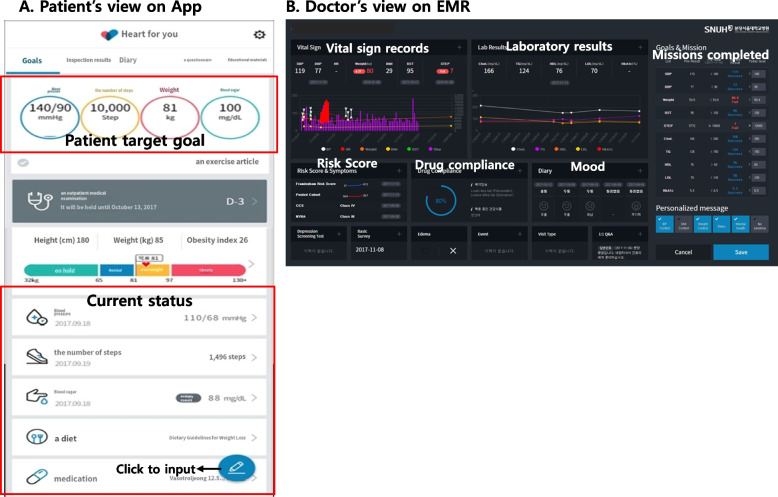
Heart4U Application. A view of the Heart4U application from the **A** patient’s perspective in the smartphone application and **B** doctor’s perspective on an electronic medical record (EMR) system

The benefits of mobile health (mHealth) for blood pressure management are well-established [10]. Our team has prior experience in developing and implementing the Heart4U application (Fig. 1), a hospital-integrated cardiovascular disease management application [11]. This application has been successfully applied in cardiovascular patients, demonstrating that its active utilization can effectively lower blood pressure and mitigate the risk of cardiovascular disease.

Given that expectant mothers nowadays are typically familiar with smartphone applications [12], we aspire to actively involve this demographic in proactive blood pressure management. Through the adoption of the Heart4U application, our research endeavors to provide active support for enhanced blood pressure control in the context of HDP, thus improving the outcomes associated with this condition. This study, aligning the convenience of technology with the challenge of managing HDP, aims to positively affect the health and well-being of mothers and infants.

### Objectives {7}

#### Research hypothesis

Through proactive blood pressure monitoring, better compliance with blood pressure measurement will result in improved blood pressure control. Additionally, healthcare providers will have the capability to promptly access and review this data within the electronic medical records system during clinical consultation, facilitating more effective patient care.

#### Primary objective

The primary objective of this study is to evaluate the difference in systolic blood pressure (SBP) measured between the point of enrollment and the conclusion of the study, which is set at 1 month ± 1 week after childbirth.

#### Secondary objectives

1. To track the trajectory of blood pressure (systolic, diastolic, and mean) within the study duration.
2. To assess obstetric outcomes, including:
  Utilization of antihypertensive medication.
  Progression to severe preeclampsia or eclampsia.
  Incidence of pulmonary edema.
  Occurrence of fetal growth restriction.
  Presence of oligohydramnios.
  Occurrence of preterm placental abruption.
  Rate of preterm delivery (between 20 and 37 weeks).
  Incidence of miscarriage (before 20 weeks).
  Frequency of fetal demise.
  Evaluation of newborn weight.
  Assessment of low birth weight.
  Incidence of postpartum hemorrhage (including transfusion).

### Trial design {8}

**Fig. 2:**
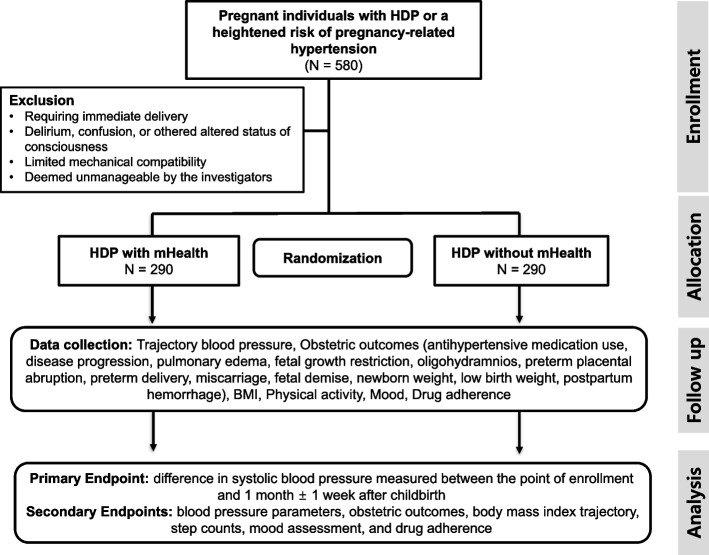
Study flowchart. A flowchart of the study protocol. HDP, hypertensive disorders of pregnancy; mHealth, mobile health

This prospective study is a randomized, single-center, open-label pragmatic trial, employing a parallel, 1:1 ratio allocation group, and superiority framework (Fig. 2). The study aims to enroll 580 pregnant individuals with HDP and comprised two intervention groups. The control group will receive standard care, while the comparison group will receive standard care along with the use of Heart4U application for proactive blood pressure measurement. Observations will be conducted in accordance with the antenatal examination schedule. Additionally, clinical follow-up will be conducted for 1 month ± 1 week after childbirth.

## Methods: participants, interventions, and outcomes

### Study setting {9}

Our study takes place at Seoul National University Bundang Hospital, a tertiary medical institution situated in the suburban area of Gyeonggi Province, South Korea. The hospital boasts a substantial capacity of 1400 beds, catering to a diverse range of medical needs. Within out institution, the obstetrics and gynecology department operates a specialized high-risk maternal and neonates in Gyeonggi Province since 2016. Annually, our center facilitates over 1200 births, with approximately 70% of patients classified as high-risk pregnancies. This significant proportion underscores our advanced capabilities in managing complex maternal health issues. Notably, more than 20% of these high-risk cases involve pregnancies complicated by hypertensive disorders, highlighting the relevance and significance of our study. One distinguishing feature of our center is the well-established collaborative system between the obstetrics and cardiology departments, particularly in the management of pregnant women with high blood pressure.

### Eligibility criteria {10}

#### Inclusion criteria

Our prospective study plans to enroll 580 pregnant individuals who meet one of the following criteria for HDP [13].

1. Chronic hypertension, defined as diagnosed hypertension within the first 20 weeks of gestation (elevated office or clinic blood pressure ≥ 140/90 mmHg) or ongoing antihypertensive medication usage
2. Gestational hypertension which involves pregnant individuals newly diagnosed with hypertension after the 20 weeks of gestation (elevated office or clinic blood pressure ≥ 140/90 mmHg) or those receiving antihypertensive treatment.
3. Preeclampsia, characterized by new-onset hypertension after 20 weeks of gestation (elevated office or clinic blood pressure ≥ 140/90 mmHg) along with concurrent indicators such as proteinuria, thrombocytopenia, abnormal renal function, elevated liver enzymes, neurological symptoms, and pulmonary edema.
4. Superimposed preeclampsia in pregnant individuals diagnosed with hypertension within 20 weeks of gestation or those on antihypertensive medication who experience symptoms or signs associated with this condition.
5. Individuals with a heightened risk of pregnancy-related hypertension due to factors such as multiple pregnancies, obesity, diabetes, or a history of hypertension-related disorders in prior pregnancies leading to preterm birth.
6. Prospective participants should be 18 years or older, possess a smartphone, be capable of using applications, and be proficient in the Korean language.

#### Exclusion criteria

Several exclusion criteria have been established to ensure the safety and feasibility of this study.

1. Pregnant individuals requiring immediate delivery due to severe preeclampsia or eclampsia are excluded.
2. Individuals displaying evidence of conditions such as delirium, confusion, or other altered states of consciousness, which can significantly impact a participant’s ability to provide informed consent, adhere to the study protocol, and accurately report outcomes.
3. Patients for whom the research process would be deemed unmanageable by the investigators.
4. Individuals with significantly limited mechanical compatibility that would impede data collection are excluded from the study to ensure that participants possess the necessary capability to independently use the application.

### Who will obtain informed consent? {26a}

Our trained research nurse and investigators provide oral and written information to the pregnant individuals with HDP. The patients can pose questions to the researchers and are afforded time to reflect upon the information before providing written consent. The nurse then collected written consent from the patients who are willing to participate. All sample information sheets and consent forms used are available in the Korean language (Supplementary file). No identifying images or other personal or clinical details of participants are presented here or will be presented in reports of the trial results. The participant information materials and informed consent form are available from the corresponding author upon request.

## Interventions

### Explanation for the choice of comparators {6b}

The participants follow the 2022 Korean Hypertension Treatment Guidelines [14]. These guidelines recommend ambulatory blood pressure monitoring for pregnant individuals with hypertension to avoid unnecessary medication. Pharmacological treatment is initiated for severe hypertension (≥ 160/110 mmHg), aiming to lower blood pressure below 150/100 mmHg without reducing diastolic pressure below 80 mmHg excessively. Postpartum, efforts are made to maintain blood pressure below 140/90 mmHg. The choice of medication considers prior usage, side effects, and fetal risk.

### Intervention description {11a}

All study participants receive the standard care and continue to receive the same proactive treatment. They are provided with OMRON automatic blood pressure monitors and are instructed by the research nurse to measure their blood pressure once daily in a quiet environment; after a minimum of 5 min of rest, participants will undergo three consecutive blood pressure measurements, each taken at 5-min intervals. The mean of the second and third measurements is recorded. Based on the measured blood pressure, if necessary and at the discretion of the treating physician, adjustments or new prescriptions for antihypertensive medications will be made.

In the intervention group, participants also receive education from the research nurse on installing and navigating the Heart4U application. At the registration point and every 2 weeks thereafter, the usage and input levels of all participants will be monitored. Participants who log in less than three times in a 2-week period will be contacted to address any issues they might face with application usage. Furthermore, in the group using the Heart4U application, medical professionals will review the information recorded in the app through the integrated electronic medical records during antenatal visits. Both groups undergo observations in accordance with the antenatal examination schedule, and the clinical follow-up period will be 1 month after childbirth.

### Criteria for discontinuing or modifying allocated interventions {11b}

Participants have the autonomy to discontinue their involvement in the trial at any juncture without needing to provide further explanations. In the event of participant withdrawal, missing data will be handled through inverse probability weighting and worst-case imputation schemes as part of the sensitivity analyses. Additionally, no crossover from the control arm to the intervention arm will be permitted during the follow-up period. Furthermore, even in the event of crossover from the control arm to the intervention arm, follow-up will continue as per the intention-to-treat (ITT) analysis protocol.

### Strategies to improve adherence to interventions {11c}

Adherence in this study is determined by the frequency of blood pressure measurements and the regularity of logging into the Heart4U application in the intervention group. The study team monitors adherence during clinical visits scheduled for antenatal examinations. To enhance adherence, the research nurse conducts regular assessments during clinical visits, ensuring that participants encounter no difficulties with using the blood pressure monitor and the application. Additional education is provided as needed to address any challenges and optimize participant engagement with the intervention.

### Relevant concomitant care permitted or prohibited during the trial {11d}

No relevant concomitant care is specifically permitted or prohibited. Managing blood pressure in pregnant individuals can be challenging due to the potential impact on utero-placental circulation and the risk of fetal anomalies associated with various medications. Given these considerations, there are typically no additional restrictions imposed on concomitant care in this trial to ensure that participants receive comprehensive and appropriate management for their HDP.

### Provisions for post-trial care {30}

No provisions are anticipated for post-trial care.

### Outcomes {12}

#### Primary outcome

The primary endpoint of this study is the difference in SBP measured between the point of enrollment and the conclusion of the study, which is 1 month ± 1 week after childbirth.

#### Secondary outcomes

1. Trajectory blood pressure: Throughout the study duration, trajectories of systolic, diastolic, and mean blood pressure will be tracked.
2. Obstetric outcomes: This includes but is not limited to:
  Antihypertensive medication use
  Progression to severe preeclampsia or eclampsia
  Pulmonary edema
  Fetal growth restriction
  Oligohydramnios
  Preterm placental abruption
  Preterm delivery (between 20 and 37 weeks)
  Miscarriage (before 20 weeks)
  Fetal demise
  Newborn weight
  Low birth weight
  Postpartum hemorrhage (including transfusion)
3. Trajectory of body mass index (BMI): analysis of BMI trajectory.
4. Physical activity monitoring: step counts recorded through Samsung Health or iPhone-Health applications.
5. Depressive Mood Assessment: depressive symptoms will be assessed using surveys, including the Patient Health Questionnaires-9 and Beck Depression Inventory [15, 16], administered at enrollment and at the conclusion of the study. These tools are widely recognized and validated measures for evaluating depression symptoms and severity.
6. Drug adherence: evaluation of medication adherence through pill counts

#### Rationale for outcomes

Our research aims to provide active support for enhanced blood pressure control in the context of HDP, thereby improving outcomes associated with this condition. By aligning the convenience of technology with the challenge of managing HDP, this study endeavors to positively affect the health and well-being of both mothers and infants. Through comprehensive monitoring and assessment of various outcomes, including blood pressure trajectories, obstetric outcomes, physical activity levels, mood, and medication adherence, we seek to gain insights into the effectiveness of our intervention in mitigating the adverse effects of HDP and promoting overall maternal and fetal health.

### Participant timeline {13}

**Fig. 3:**
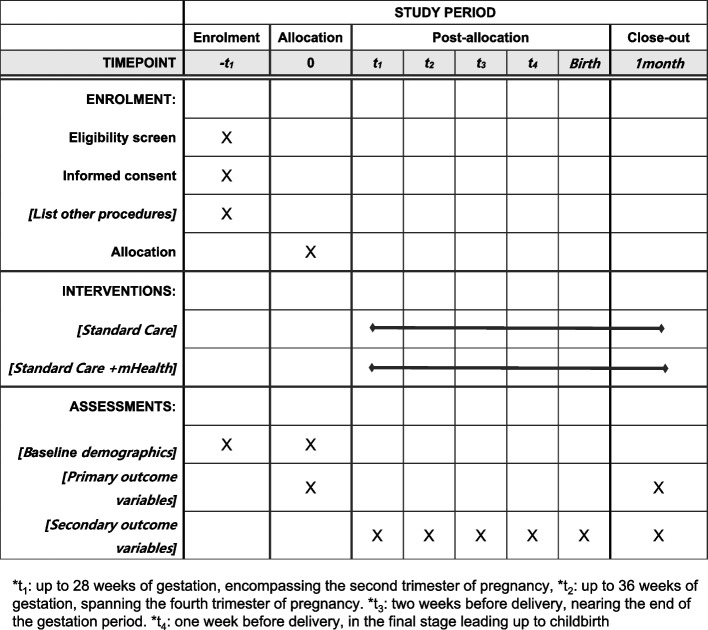
Timeline for participants

The participant timeline is shown in Fig. 3.

### Sample size {14}

The determination of the sample size for this study, with an intended enrollment of 580 participants, was established using specific parameters as follows [17, 18]: the mean SBP at the point of enrollment was 146 mmHg, accompanied by a standard deviation of 13 mmHg. Subsequently, the mean SBP 1 month after childbirth was projected to be 130 mmHg, with a corresponding standard deviation of 12 mmHg. The control group displayed a delta SBP of 16 mmHg, whereas the delta SBP of the intervention group was amplified by 20% to 19.2 mmHg. The required difference between the two groups for sample size computation was 3.2. Moreover, a standard deviation of 13 mmHg was attributed to the delta SBP. These considerations are further coupled with an alpha error of 0.05 and a power of 80%. Using these variables, the calculations stipulated the necessity of 261 participants within each group, culminating in a total of 522. Considering a presumed dropout rate of 10%, the required comprehensive sample size was 580 participants.

### Recruitment {15}

Recruitment for our trial targets pregnant individuals diagnosed with HDP who are attending our hospital’s high-risk maternal care center. Screening for eligibility criteria is conducted by the attending obstetrician, who verifies whether the patients meet the specified criteria. Patients receive a comprehensive explanation of the trial’s risks and benefits to ensure their full understanding before being asked to provide voluntary written consent for participation. This process aims to ensure that participants are fully informed and willing to enroll in the study.

## Assignment of interventions: allocation

### Sequence generation {16a}

In this study, the allocation sequence was generated using SAS version 9.4 (SAS Institute, Cary, NC, USA) software. The allocation sequence was implemented and managed in the web-based case report form (CRF) system provided by GoodResearchUS (Seoul, Korea), where system roles and access permissions are established. Stratified permuted-block randomization was employed to ensure balanced assignment of subjects to each group. Block sizes were determined as multiples of 2 (e.g., 2, 4, 6). These blocks were generated using stratified randomization, with medication adherence as a stratification factor. Upon patient enrollment, the web-based CRF system generates a randomization table, automatically assigning participants to either the intervention or control group. This process is centrally managed, and allocation information is disclosed to participants immediately after group assignment.

### Concealment mechanism {16b}

Random allocation is achieved using a centralized, web-based randomization system based on a pre-prepared randomization table. The randomization table is not disclosed to the researchers and is securely stored in the web-based CRF database managed by the vendor. Upon participant registration, the randomization table generation function automatically assigns participants to either the intervention or control group. This process rigorously conceals the allocation sequence to ensure the reliability of the study. Allocation information is disclosed to participants only immediately after group assignment, maintaining allocation concealment throughout the study.

### Implementation {16c}

Enrollment of participants will be conducted by sub-investigators, including obstetric physicians who are blinded to the allocation. The randomization module will be utilized to assign participants without disclosing the complete allocation scheme. This approach ensures that the enrollment process remains unbiased and maintains the blinding of both participants and investigators throughout the study.

## Assignment of interventions: blinding

### Who will be blinded {17a}

Blinding of the participants is not feasible due to the nature of the intervention in this trial. Additionally, the attending physicians cannot be blinded as they need to share blood pressure monitor data and provide feedback to patients in the intervention group. However, outcome assessors will be blinded to ensure that their assessments do not influence the analysis results.

### Procedure for unblinding if needed {17b}

The design is open label with only outcome assessors being blinded so unblinding will not occur.

## Data collection and management

### Plans for assessment and collection of outcomes {18a}

For the assessment and collection of outcomes, baseline information will be collected by trained research nurses who will input the data into a web-based CRF at the time of study registration. Both groups will receive an OMRON blood pressure monitor, with the standard care group having their self-measured blood pressure automatically entered into the OMRON application via Bluetooth. In contrast, the group using Heart4U will have their measured blood pressure automatically synchronized with the application. The synchronized data will be seamlessly integrated with the web-based CRF, eliminating the need for additional data collection. Any errors or discrepancies in the data will be managed by an independent data committee, which is designed to notify stakeholders of any incorrect values.

### Plans to promote participant retention and complete follow-up {18b}

Participants in this study have the autonomy to withdraw from the research at any point without facing any obligation to complete the study. As a pragmatic trial grounded in real-world settings, there is no compulsion for participants to fulfill additional follow-up requirements beyond their routine antenatal examinations and the standard postpartum outpatient visit scheduled 1 month after childbirth. Given that data collection aligns with participants’ regular antenatal examination schedules and the final follow-up coincides with the typical postpartum outpatient visit, we anticipate minimal participant burden and expect the dropout rate to be low. This approach aims to integrate the research seamlessly into participants’ existing healthcare routines, promoting retention and adherence throughout the study duration.

### Data management {19}

In our study, we utilize a web-based CRF system to facilitate direct digital data entry, standardize data collection processes, and automate data management. This approach enhances the accuracy of data, reduces input errors, and streamlines data processing time. Additionally, our system incorporates automated queries within the web-based CRF to identify and address missing data or inconsistencies, ensuring data integrity.

Following data collection, we perform thorough data validation and cleaning processes to review the consistency, accuracy, and safety of the data. Continuous access to the web-based CRF and Excel files allows for ongoing data review and potential modifications as needed. This iterative process ensures the quality and reliability of the data throughout the study duration. Overall, our data management approach ensures standardized, efficient, and secure handling of data, ultimately contributing to the quality assurance of our study outcomes.

### Confidentiality {27}

All people involved in the study are subject to confidentiality. All data will be collected and analyzed pseudonymously. Extensive measures will be taken to protect the data, especially personal data, against access by third parties. Local therapy files and study data will be stored in locked cabinets and will only be accessible to study staff and to the respective treating therapists who are subject to the legal duty of confidentiality. The data collected so far will further be used and analyzed within the study, unless participants withdraw their consent to processing their data as well. If a participant only discontinues the study treatment, but not participation in general, further data required for the study can be collected and used.

All therapists, supervisors, and study assistants are also subject to medical confidentiality. No information collected during the therapy will be passed on to third parties unless the participants give their explicit written consent.

### Plans for collection, laboratory evaluation, and storage of biological specimens for genetic or molecular analysis in this trial/future use {33}

Not applicable as no biological specimens will be collected.

## Statistical methods

### Statistical methods for primary and secondary outcomes {20a}

All continuous variables among the primary and secondary efficacy evaluation variables will be presented as mean and standard error or median and quartiles, and categorical variables will be summarized as percentages compared with the population parameters. Normally, two-sample *t*-tests or the Mann–Whitney *U* test, depending on whether the data follow a normal distribution, are used for continuous variables after assessing normality using the Kolmogorov–Smirnov test. The distributions of categorical variables are analyzed with *χ*^2^ tests or Fisher’s exact tests. The ITT principle will be used for all analyses. For each subgroup, we will calculate the hazard ratio and 95% confidence intervals for the primary and secondary endpoints. Statistical significance is set at a two-sided *P*-value of < 0.05. Statistical analyses will be performed using R version 4.3.1 (R Foundation for Statistical Computing, Vienna, Austria). The datasets analyzed in the current study will be available from the corresponding author upon request.

### Interim analyses {21b}

Not applicable since there will be no interim analyses regarding the main objective of the trial.

### Methods for additional analyses {20b}

In addition to the primary and secondary analyses outlined in the study protocol, we plan to conduct additional analyses to explore the treatment effect based on the frequency of application use as derived from log data. The treatment effect will be compared across different categories of application use, including weekly, biweekly, monthly, bimonthly, other, and no use. Specifically, “Weekly” will be defined as at least one use every week, while “other” will indicate less than one use in 2 months. “No use” will indicate zero or one use during the overall study period.

These additional analyses aim to provide insights into the relationship between the frequency of application use and treatment outcomes, allowing for a more nuanced understanding of the impact of mHealth intervention on blood pressure management in pregnant individuals with HDP.

### Methods in analysis to handle protocol non-adherence and any statistical methods to handle missing data {20c}

In line with our study’s pragmatic approach and aim to assess real-world effectiveness, we have planned the analysis using the ITT principle. This approach allows us to evaluate the intervention effect while considering all participants according to their assigned group, regardless of protocol adherence.

In our study, which focuses on evaluating the effectiveness of a non-invasive intervention using a mobile health application rather than treatment, assessing non-adherence involves measuring compliance through log data generated by the mobile health application. We plan to conduct a sub-analysis specifically dedicated to examining compliance based on this log data. This approach allows us to quantify and evaluate non-adherence objectively.

For handling missing data, we will investigate the reasons behind their occurrence and determine whether they are independent of the outcome. Additionally, efforts will be made to retrieve lost information for patients who have not withdrawn consent. Multiple imputation methods will be prioritized over simple data imputation techniques due to their ability to generate imputations using regression models and create multiple imputed datasets, which helps reduce bias and improve variability estimation. Data imputation will be deemed reasonable under specific conditions, such as when the frequency of missingness is low, the outcome variable holds clinical significance, and viable strategies for imputation are available.

### Plans to give access to the full protocol, participant-level data, and statistical code {31c}

The full study report, the anonymized data set, and the statistical code will be made available from the corresponding author upon reasonable request after the main results have been published and as long as it corresponds with the local regulations for data.

## Oversight and monitoring

### Composition of the coordinating center and trial steering committee {5d}

This study is conducted at a single site, eliminating the need for coordination between multiple centers. As a result, there is no independent oversight committee involved in monitoring the trial. However, the Principal Investigator (PI), Jung-Won Suh, will hold regular meetings with the management team, co-investigators, and the data and safety monitoring board to ensure effective management and oversight of the trial.

### Composition of the data monitoring committee, its role, and reporting structure {21a}

The Data Monitoring Committee (DMC) for this study consists of three members experienced in cardiology, obstetrics and gynecology, and data analysis. These experts are from the same institution and have direct access to electronic medical records, enabling efficient communication for resolving discrepancies and troubleshooting issues. They are blinded and unbiased, ensuring impartial review of suspected clinical endpoints and adverse events.

The role of the DMC is to conduct a thorough review of suspected clinical endpoints and adverse events. Their objective is to standardize the review process for clinically relevant endpoints and minimize bias and variability from investigators involved in the study. As independent members, they operate free from any influence or regulations by the PI.

The reporting structure involves regular meetings between the DMC members to discuss and evaluate the data collected from the study. They provide recommendations and insights to the PI and the study management team based on their findings, contributing to the overall monitoring and oversight of the trial.

### Adverse event reporting and harms {22}

Given the nature of the intervention in this trial, no adverse events are anticipated to result directly from the intervention. The study involves self-monitoring through a mobile application, which does not entail invasive or harmful procedures. Additionally, as an open-label study, continuous monitoring by the attending physician throughout the trial ensures prompt identification and management of any potential concerns, minimizing associated risks.

However, it is crucial to acknowledge the potential risks inherent in using mobile health applications, particularly concerning privacy and security. To address these concerns, several measures are implemented. Firstly, data collection is conducted primarily using de-identified data to safeguard participant privacy. Furthermore, researchers collect only the minimum necessary data required for the research, reducing the risk of re-identification. Robust security protocols, including encryption and access controls, are employed to protect against unauthorized access or disclosure of sensitive information.

To mitigate the risk of misinterpretation due to inaccurate or incomplete information, the DMC is responsible for the meticulous review of all data collected, providing recommendations to the management team to ensure the integrity and accuracy of the study findings. This proactive approach enhances transparency and rigor in data interpretation, further safeguarding participant well-being and study integrity.

### Frequency and plans for auditing trial conduct {23}

The DMC has agreed to meet at the conclusion of each data collection period to review the trial conduct. Additionally, they will inform the research team of any issue as they arise. Furthermore, the research team will convene every 2 weeks for a trial review.

### Plans for communicating important protocol amendments to relevant parties (e.g., trial participants, ethical committees) {25}

Important protocol modifications were communicated to the research ethics committee and, if necessary, to the trial participants.

### Dissemination plans {31a}

The trial results will be disseminated nationally and internationally to reach participants, academic researchers, and the public upon completion of the trial. Dissemination activities will include scientific presentations at conferences and publication in peer-reviewed journals. However, to uphold the scientific integrity of the trial, data will not be released for publication or oral presentation purposes before the end of the trial without the permission of the PI.

## Discussion

HDP is recognized to be closely related to adverse pregnancy outcomes [19, 20], and its impact extends beyond maternal health and has implications for postpartum cardiovascular outcomes [21]. Therefore, the proactive management of HDP is of paramount importance. However, the current landscape is limited by fetal safety issues, which constrain pharmaceutical interventions within this context [7, 8]. Although addressing HDP is essential for both maternal and fetal well-being, a cautious approach to medication underscores the complexity of balancing therapeutic benefits with potential risks to the developing fetus [22].

Recognizing advancements in technology, hypertension management extends beyond conventional pharmaceutical interventions. Self-management of hypertension through platforms such as mHealth have emerged, enabling patients to actively monitor and regulate their blood pressure while facilitating communication with healthcare providers [23–25]. In addition to traditional pharmacotherapy, the integration of innovative tools such as mHealth has demonstrated the potential to achieve proactive blood pressure control and, in certain studies, even yield improvements in cardiovascular outcomes [10].

This study was meticulously designed to capitalize on the advantages of mHealth to effectively support blood pressure control in individuals with HDP, a population in which the availability of pharmaceutical interventions may be limited. Through the utilization of the Heart4U application, we aspire to enhance participant awareness of their blood pressure status and facilitate seamless communication with healthcare providers. This approach serves as a dual-fold strategy. First, by empowering proactive blood pressure management, we seek to amplify individual engagement in health outcomes. Second, we anticipate that this endeavor will translate into improved maternal and fetal outcomes, ultimately extending to a potential influence on postpartum outcomes as well.

By embracing a non-invasive intervention model that employs smartphones, a tool familiar to most individuals, this study strives to foster regular health monitoring. The Heart4U application, integrated with medical services, not only facilitates effective communication with health professionals, but also allows for convenient follow-up outside the clinical setting. As a result, we anticipate minimal adverse effects, while expecting to reap a multitude of benefits. This innovative approach uses modern technology to empower patients, augment awareness, enhance communication, and potentially trigger positive health outcomes.

In conclusion, this study highlights the significance of proactive management for HDP and introduces the innovative potential of mHealth solutions, such as the Heart4U application. This study aims to investigate a pathway for improving maternal, fetal, and postpartum outcomes in patients with HDP by enhancing blood pressure control and facilitating effective communication. Our study seeks to provide valuable insights into the effectiveness of these interventions, contributing to the advancement of maternal healthcare practices.

## Trial status

Publication based on Protocol V1.0, 16 Aug 2023. The study center is open to recruitment starting from September 2023. Recruitment is anticipated to conclude by August 2026, with the study expected to be fully completed, including data collection, by June 2027.

### Supplementary Information

Supplementary Material 1.

Supplementary Material 2.

## Data Availability

The full study report, the anonymized data set, and the statistical code will be made available from the corresponding author upon reasonable request.

## Abbreviations

HDP: Hypertensive disorders of pregnancy
mHealth: Mobile health
SBP: Systolic blood pressure
BMI: Body mass index
CRF: Case report form
ITT: Intention-to-treat
PI: Principal investigator
DMC: Data monitoring committee
