# Supplementary material for: Prospective validation of a mobile health application for blood pressure management in patients with hypertensive disorders of pregnancy: study protocol for a randomized controlled trial
Source: Trials. 2024 Jul 2;25:435. doi: 10.1186/s13063-024-08200-y (PMC11218072; doi:10.1186/s13063-024-08200-y)
Supplement: Supplementary file 1 — Supplementary Material 1. [file 13063_2024_8200_MOESM1_ESM.docx]

**Subject Information Consent Sheet (V1.0) (V1.0)**

Screening Number of the Subject:________________

Title of the Study: Validation of the Utility of Managing Pregnant Women with Hypertension through Hospital Clinic-Linked Cardiovascular Disease Management Service Solution

Principal Investigator: Professor Jung-won, Suh, Department of Cardiology

We kindly request your participation in this study. It is important that you fully understand why this study is being conducted and what will be involved before deciding to participate. The following information is provided to explain the nature of this study, your role in it, and the procedures involved. Please take ample time to read through this subject information sheet, and feel free to discuss it with your family or others. Additionally, if you have any questions, please consult with the principal investigator or other research staff before making a decision to participate in this study.

1. Purpose of the Study This clinical research is not for therapeutic purposes but is conducted to evaluate the effectiveness of self-management through the cardiovascular disease management app (Heart4U) and to better understand cardiovascular diseases.
2. Background and Objective of the Clinical Study The purpose of this study is to evaluate the clinical efficacy of a treatment strategy for managing risk factors using the smartphone application (Heart4U) among pregnant women with hypertension who require ongoing monitoring.
3. Information on Investigational Drugs/Medical Devices for the Clinical Study and Randomization Probability

- App Group: Pregnant women with hypertension assigned to the App Group will receive assistance from the research team to install and familiarize themselves with the Heart4U app.
- Usual Care Group: Active treatment for pregnancy-induced hypertension based on guidelines. Both the App Group and Usual Care Group will receive the same active treatment for pregnancy-induced hypertension as before (guideline-based prenatal care).

This study is a randomized double-blind study. Your assignment to either group will be determined randomly by a computer-generated randomization table, with an equal probability of 1:1.

**The participants in the clinical study will undergo various tests and procedures.**

During the clinical study period, various tests and procedures will be conducted, including several surveys and questionnaires, which will be administered as part of routine outpatient visits. For blood tests, no additional procedures will be conducted specifically for the clinical trial; instead, we will refer to the results of tests conducted during outpatient visits (medical records).

| **Time** | **Exam and interventions** |
| --- | --- |
| At registration | - Vital signs assessment (blood pressure), Anthropometric measurements (height, weight), Blood sampling (for glucose, cholesterol levels), Inquiry about current medication usage (including within the past month), Assessment of smoking status, Inquiry about the use of antihypertensive medications - Additionally, participants will complete surveys regarding: Physical activity, Depression, Medication adherence |
| Prenatal visits | - Vital signs assessment (blood pressure), Height, Body weight - Inquiry about current medication usage - obstetric complications - information recorded in the app |
| Postpartum  (1mo ±1wk)  (Final visit) | - Vital signs assessment (blood pressure), Height, Body weight, blood test(DM, dyslipidemia), Inquiry about current medication usage   Inquiry about the use of antihypertensive medications, smoking   - information recorded in the app - Survey (Physical activity, Depression, Medication adherence) |

**Responsibilities for Participants**

If you decide to participate in this clinical study, please adhere to the following:

- Cooperate with blood pressure measurements taken at each obstetrics and gynecology outpatient visit.
- Respond honestly to the surveys conducted at each visit.
- Inform the researchers immediately if there are any changes in your health or if you have any concerns about the clinical study.

Acknowledgment of an Unproven Clinical Study:

Not applicable.

**Potential Side Effects, Risks, or Discomforts for Participants:**

There are no anticipated side effects, risks, or discomforts associated with participating in this study. However, if you experience any minor discomfort during the study period, please inform the researchers promptly.

**Expected Benefits for Participants:**

Participating in this study may not directly benefit you. However, the information obtained from your participation may provide valuable insights for other patients in similar situations.

**Alternative Treatments (Other Available Treatment Options):**

Not applicable.

**Injury and Compensation:**

While no injuries are expected to occur as a result of the study, if any serious adverse reactions occur, appropriate measures will be taken promptly to minimize them. If you experience any harm related to participating in the study, you will be treated with the best possible methods.

**Financial Compensation:**

Participants in this study will be provided with a Bluetooth-enabled automatic blood pressure monitor.

**Expected Costs:**

There will be no additional costs incurred by participating in this clinical study.

**Voluntary Participation:**

Participation in this clinical study is entirely voluntary. You are free to refuse participation after hearing the explanation of the study. Furthermore, even after consenting to participate, you may withdraw from the study at any time without any consequences or disadvantages.

**Provision of Personal Information:**

During and after the conduct of the study, the monitor of the clinical study, inspectors, ethics committees, the Minister of Food and Drug Safety, the Minister of Health and Welfare, etc., may directly access your medical records and other research-related data within the scope prescribed by regulations, without violating the confidentiality of your information. By signing the consent form, you or your representative allow the direct access of such data. Additionally, if you do not consent to the provision of your personal information to third parties, it will not be shared.

**Confidentiality:**

All records of your personal information obtained during the study will be kept confidential and will not be disclosed to others. Even when the research results are published, your personal information will remain confidential.

**Ongoing Provision of Information Related to the Study:**

During the course of the study, if any new facts or information come to light that may affect your decision to continue participating, the researcher will inform you or your representative of this fact or information promptly..

**Contact Information:**

You or your representative can schedule a phone consultation with the following individuals:

For issues, concerns, or questions related to the clinical study:

Principal Investigator: Professor Suh Jung-won, Department of Cardiology ☎031-787-7016

Research Coordinator: Researcher Kim Hye-jin, Department of Cardiology ☎010-7408-2523

For issues, concerns, or questions regarding the rights of research participants: Office of the Institutional Review Board (IRB) ☎ 031-787-8801~8806

Clinical Research Ethics Center ☎031-787-8811~8813

**Termination of the Study:**

Your attending physician may discontinue your participation if you fail to comply with the instructions or for medical or other reasons. Additionally, the study may be terminated if it is determined that continuing the study does not provide any benefits to you based on your opinion or if any significant adverse reactions occur.

**Expected Duration of Participant Involvement in the Study:**

By participating in this study, you will undergo a research period of approximately 10 months (until childbirth ±1 month).

**Approximate Number of Participants in the Study:**

A total of 580 participants will be involved in this study.

- **Other:**
- After hearing the introduction to this study, if you decide to participate, please sign and date the consent form provided.
- In cases where the participant is unable to provide consent due to circumstances such as lack of understanding or inability to express consent, consent must be obtained from a legal guardian or conservator. In such cases, the researcher and research staff must provide information about the study to the participant to the extent they can understand, and if possible, the participant should sign and date the consent form in their own handwriting.
- If the participant or their representative is unable to read the consent form, participant observation is required for all consent processes.
- One copy of the explanation sheet and the consent form that you sign will be provided to you.

**연구대상자 동의서**

**연구의 제목:** 병원진료 연계 기반의 심혈관 질환 관리 서비스 솔루션을 통한 임신성 고혈압 환자 관리 유용성 검증`

1. 나는 본 연구에 대한 모든 정보들에 관하여 담당의사로부터 자세하게 설명을 듣고 충분히 이해 하였습니다.
2. 나는 또한 대상자 동의설명서를 읽어 보았으며, 그 내용을 충분히 이해하였으며 본 연구가 연구 목적으로 수행된다는 사실을 알고 있습니다.
3. 나의 연구 참여여부 결정은 자발적인 것이며, 연구 기간 중 언제라도 개인적인 사유 등으로 지속적인 참여를 중도에 거부하거나 자유로이 참가를 중단할 수 있으며, 이로 인해 진료 및 기타 어떠한 불이익도 받지 않음을 알고 있습니다.
4. 나는 연구 관련하여 의문이 있을 경우에는 언제라도 연구자에게 문의할 수 있으며, 나의 의무기 록을 연구 목적으로만 직접 열람하는 데에 동의합니다.

이에 나의 자유로운 의사에 따라 본 연구에 참여할 것을 동의합니다.

|  | **성 명** | **서 명** | **서 명 날 짜** |
| --- | --- | --- | --- |
| **대상자** |  |  | 년 월 일 |
| **대상자의 대리인**  (이해능력∙의사표현 능력의 결여 등인 경우만 해당) |  |  | 년 월 일 |
|  | 대상자와의 관계: ________________________  구체적인 사유: _____________________________________________________________ | | |
| **연구책임자**  **(또는 공동연구자)** |  |  | 년 월 일 |

해당되는 경우 (문서화된 정보를 읽을 수 없는 경우)

|  | **성 명** | **서 명** | **서 명 날 짜** |
| --- | --- | --- | --- |
| **참관인** |  |  | 년 월 일 |
